# MR Imaging of Pediatric Brain Tumors

**DOI:** 10.3390/diagnostics12040961

**Published:** 2022-04-12

**Authors:** Alok Jaju, Kristen W. Yeom, Maura E. Ryan

**Affiliations:** 1 Department of Medical Imaging, Ann and Robert H. Lurie Children’s Hospital of Chicago, Northwestern University Feinberg School of Medicine, Chicago, IL 60611, USA; mryan@luriechildrens.org; 2 Department of Radiology, Lucile Packard Children’s Hospital, Stanford University, Palo Alto, CA 94304, USA; kyeom@stanford.edu

**Keywords:** pediatric brain tumors, MRI, advanced imaging, medulloblastoma, embryonal tumors, pediatric glioma, juvenile pilocytic astrocytoma

## Abstract

Primary brain tumors are the most common solid neoplasms in children and a leading cause of mortality in this population. MRI plays a central role in the diagnosis, characterization, treatment planning, and disease surveillance of intracranial tumors. The purpose of this review is to provide an overview of imaging methodology, including conventional and advanced MRI techniques, and illustrate the MRI appearances of common pediatric brain tumors.

## 1. Introduction

Primary brain tumors are the most common solid neoplasms in children and a leading cause of mortality in this population. Brain tumors have a reported incidence of 6.06 per 100,000 children 0–19 years of age in the United States [1,2].

Imaging plays a central role in the diagnosis, characterization, treatment planning, and disease surveillance of intracranial tumors. Magnetic resonance imaging (MRI) is the mainstay of neuroimaging and provides anatomical details, as well as cellular, vascular, and functional information for brain tumors. Imaging features, in combination with location, demographics, and clinical presentation, can help arrive at an accurate diagnosis or a narrow differential diagnosis. The purpose of this review is to provide an overview of imaging methodology, including conventional and advanced MRI techniques, and illustrate the MRI appearances of common or characteristic pediatric brain tumors. 

For this review, tumors are organized according to the 2021 World Health Organization classification of Central Nervous System tumors (CNS WHO5) [3], highlighting important distinctions between major tumor groups, including embryonal tumors, pediatric-type diffuse high-grade gliomas, pediatric-type diffuse low-grade gliomas, circumscribed astrocytic gliomas, ependymal tumors, glioneuronal or neuronal tumors, choroid plexus tumors, and pineal region tumors.

## 2. Imaging Techniques

Due to its speed and availability, computed tomography (CT) of the head without contrast may be the initial modality for screening patients with signs and symptoms suggestive of an intracranial space-occupying lesion. CT can provide useful information about the presence of a mass, its location, associated mass effect, and hydrocephalus. CT may identify solid and cystic components, hemorrhage, and calcifications, but MRI is required for adequate tumor characterization staging. Post-contrast CT is typically of little added benefit, and a lesion initially identified on non-contrast CT should be further evaluated by MRI.

MRI can be performed on a 1.5 or a 3 Tesla scanner; however, a 3 Tesla scanner has the advantages of better signal-to-noise ratio and faster imaging. MRI study times vary but often require 30–60 min. Therefore, sedation or general anesthesia is typically needed in children younger than six years of age, although this can be assessed on a case-by-case basis. Child life specialists can be very helpful in improving patient compliance. The use of gadolinium contrast material is standard for tumor imaging, although there may be some exceptions. 

A conventional brain MRI includes pre- and post-contrast T1-weighted, T2-weighted, fluid-attenuated inversion recovery (FLAIR), and diffusion-weighted sequences. The use of high-resolution, thin section, 3-dimensional imaging, which can be reformatted in multiple planes, is now standard for T1-weighted imaging and is gaining wider acceptance for T2 and FLAIR sequences. In addition, technical advances in imaging methodology such as compressed sensing and parallel imaging allow for the acquisition of high-resolution and thinner slices with a reasonable scan time. The Response Assessment in Pediatric Neuro-Oncology (RAPNO) working groups have published guidelines for minimum essential MRI sequences for different types of pediatric brain tumors, and the reader is referred to those detailed recommendations [4,5,6,7].

Diffusion-weighted images measure the mobility of water molecules, which in turn depends on the complexity of cytoarchitecture. High-grade tumors, because of their higher cellularity and high nuclear-to-cytoplasmic ratios, demonstrate restricted diffusion, which can be measured quantitatively as decreased apparent diffusion coefficient (ADC) values [8]. Diffusion-weighted imaging is very valuable for the differential diagnosis of brain tumors as well as for the detection of leptomeningeal metastasis, particularly for non-enhancing tumors [9]. Susceptibility-weighted imaging (SWI), which is highly sensitive for paramagnetic or diamagnetic compounds and can detect areas of hemorrhage or calcifications, is also commonly included in standard brain imaging [10].

### 2.1. Advanced Techniques

Advanced imaging techniques may provide microstructural, hemodynamic, and metabolic information, and can be performed at most centers, although they do require additional imaging time and post-processing capabilities. Diffusion tensor imaging (DTI) assesses the magnitude as well as the direction of water diffusion and can be postprocessed to generate white matter tracts. DTI and tractography can be overlaid on anatomical images to provide valuable information about the relationship of the tumor to the major white matter tracts and help guide the surgical approach [11].

MR perfusion techniques provide an assessment of tumor vascularity and hemodynamics. Perfusion can be performed using contrast-enhanced techniques, such as dynamic susceptibility (DSC), dynamic contrast-enhanced (DCE) perfusion, or non-contrast arterial spin labeling (ASL) technique. Depending on the method use, MR perfusion can generate hemodynamic parameters, such as relative cerebral blood flow (rCBF), relative cerebral blood volume (rCBV), time to peak (TTP), mean transit time (MTT), and vascular permeability or transfer coefficient (K-trans). These can provide valuable insights into tumor grade, treatment response, and help in differentiating tumor from radiation injury [11]. 

Magnetic Resonance Spectroscopy (MRS) provides an analysis of the biochemical composition of the tissue and can be useful for the differential diagnosis and grading of tumors. Proton MRS is the most widely used technique and can be performed as single-voxel or chemical shift imaging (multi-voxel). High-grade tumors typically have elevated choline peaks, decreased n-acetyl aspartate (NAA) peak, and variable lipid/lactate peak. However, there are exceptions, and low-grade tumors such as pilocytic astrocytoma can demonstrate a similar spectroscopic appearance. Short TE MRS (35 milliseconds) allows the detection of additional metabolite peaks, such as myo-inositol (mI, a glial cell marker), glycine, glutamine/glutamate (Glx), taurine (Tau), alanine (Ala), and citrate (Cit), which can be further helpful in tumor characterization. MRS can also be helpful in differentiating tumors from post-treatment change [12,13].

Functional MRI (fMRI) uses regional changes in brain perfusion induced by brain activation to generate an MRI signal. This can non-invasively map various eloquent areas, such as motor, sensory, language, and visual, during the performance of specific tasks. This information can be used by neurosurgeons to plan the approach and extent of tumor resection [14].

### 2.2. Spine Imaging

Based on the suspected tumor histology, imaging of the spine may be added for the evaluation of cerebrospinal fluid (CSF) dissemination. As with intracranial disease, contrast is typically required for spine evaluation, although high-resolution 3D steady-state T2 imaging, such as constructive interference in steady state, Siemens (CISS), or fast imaging employing steady-state acquisition, GE (FIESTA-C), can be particularly helpful in identifying small lesions and non-enhancing disease along the surface of the spinal cord or cauda equina nerve roots [15]. Limited spine imaging with post-contrast T1 and 3D steady-state T2 sequences is usually adequate for routine metastatic surveillance, without the need for pre-contrast imaging, thus decreasing the total imaging and, if applicable, anesthesia time in these patients. As with intracranial disease, DWI can be a valuable technique for metastasis detection in the spine, especially for non-enhancing lesions. However, due to the relatively poor resolution of DWI in the spine, it has limited added value for routine surveillance spine MRI and is often best used as a problem-solving tool [16]. It is strongly recommended that spine MRI for initial staging in the setting of a newly diagnosed posterior fossa tumor be performed pre-operatively, as blood products and dural reactions can significantly limit interpretation in the immediate post-operative period. If the spine MRI is not performed pre-operatively, waiting for at least 2 weeks after posterior fossa surgery is recommended to avoid false positive results [17,18].

Recent advances in artificial intelligence and computer vision in medicine have opened a new frontier in neuro-oncology research. Techniques such as radiomics and deep learning have shown great promise in prospective radiological diagnosis, molecular subtype prediction, and outcome predictions for both pediatric and adult brain tumors [19,20,21].

### 2.3. Post-Operative and Surveillance Imaging

Post-operative imaging is essential to evaluate the extent of surgical resection, to identify any immediate post-operative complications, and as a baseline for assessing the response to radiation and chemotherapy. In specialized centers, intraoperative MRI may also be used to guide surgical management, with recent advances allowing the use of high-field-strength systems, diagnostic-quality images, and the ability to integrate with the surgical navigation systems, making it an important tool for neurosurgeons [22,23,24].

Post-operative MRI should be performed within 72 h of surgery and preferably within the first 24 h, as distinguishing residual tumors from reactive enhancement on delayed imaging can be challenging [25]. In the post-operative period, hemorrhage along the resection margins may demonstrate T1 shortening; therefore, a direct comparison of pre- and post-contrast images obtained in the same imaging plane is important to distinguish hemorrhage from true enhancement. DWI is a very helpful technique for both post-operative and long-term surveillance imaging. In the immediate post-operative period, restricted diffusion along the surgical margins may be related to post-surgical change, and a careful correlation of these areas with contrast enhancement on follow-up imaging should be performed to distinguish post-surgical enhancement from tumors. DWI can also be helpful for assessing residual or recurrent disease in high-grade tumors [22].

As a potential complication of treatment, radiation necrosis can occur months to decades after treatment and is characterized by areas of enhancement, T2 prolongation, and edema that can mimic tumor recurrence on conventional MR images. Advanced techniques including MR spectroscopy and MR perfusion imaging are useful in increasing the diagnostic confidence [26,27]. Another long-term complication of cranial radiation is the development of intracranial cavernomas, which can sometimes cause larger bleeds and require surgical intervention. An assessment with susceptibility-weighted images is important for monitoring this finding [28].

Olivary degeneration is a specific complication related to posterior fossa surgery and is believed to be secondary to damage to the dentato-rubro-olivary pathway. On imaging, it can manifest as T2 signal changes in the inferior olivary nuclei of the ventral medulla (with or without hypertrophy) and cerebellar dentate nuclei. These findings have been suggested as an imaging biomarker for posterior fossa syndrome, a severe clinical condition presenting as mutism, dysmetria, and ataxia [29].

## 3. Conventional and Advanced Imaging Features of Major Tumor Types

### 3.1. Embryonal Tumors

Embryonal tumors of the central nervous system are highly malignant, undifferentiated, or poorly differentiated tumors of neuroepithelial origin, designated as WHO grade 4. The classification of embryonal tumors has evolved over multiple iterations of WHO classification [3,30]. Medulloblastoma, which are exclusive to the posterior fossa, are the most common embryonal tumors, accounting for two-thirds of cases. Atypical teratoid/rhabdoid tumor (AT/RT) can occur in both supra- and infratentorial locations, although they are more common in the posterior fossa. The remaining embryonal tumors are predominantly supratentorial and replace the previously used category of primitive neuroectodermal tumors (PNET); they are subgrouped based on molecular features [31]. Key MRI features of embryonal tumors are summarized in Table 1.

### 3.2. Medulloblastoma

Medulloblastoma accounts for 25% of all intracranial pediatric tumors and is the second most common pediatric brain tumor, after pilocytic astrocytoma. It is the most common malignant pediatric CNS tumor and the most common posterior fossa tumor, comprising approximately 38% of posterior fossa tumors in children. The median age is 9 years at presentation, although nearly 25% of all medulloblastomas arise in adults. Amongst those older than 3 years, males are more commonly affected (1.7:1), while there is no gender predilection for those younger than 3 years [31].

Medulloblastomas are WHO grade 4 embryonal tumors and their MR imaging characteristics reflect a high cellularity. A key imaging feature of medulloblastoma is diffusion restriction, with DWI signal greater than that of adjacent cerebellum and corresponding low ADC values. The ADC values in medulloblastoma are usually less than 1 × 10^−3^ mm^2^/s, with mean ADC values reported from 0.5–0.7 [32,33]. These lesions typically have a rounded morphology, relatively well-defined margins, and variable T2 signal ranging from T2 hyperintense to T2 hypointense compared to the cortex. Areas of lower T2 signal is typically an indicator of greater cellularity [34]. Enhancement is also variable, ranging from robust to mild or no identifiable enhancement. Cysts or necrosis are not uncommon, estimated to be present in 40–50% of medulloblastomas, but are typically small [35]. Calcification can occur but is uncommon and not a predominant feature. Surrounding edema may be present [36].

**Figure 1:**
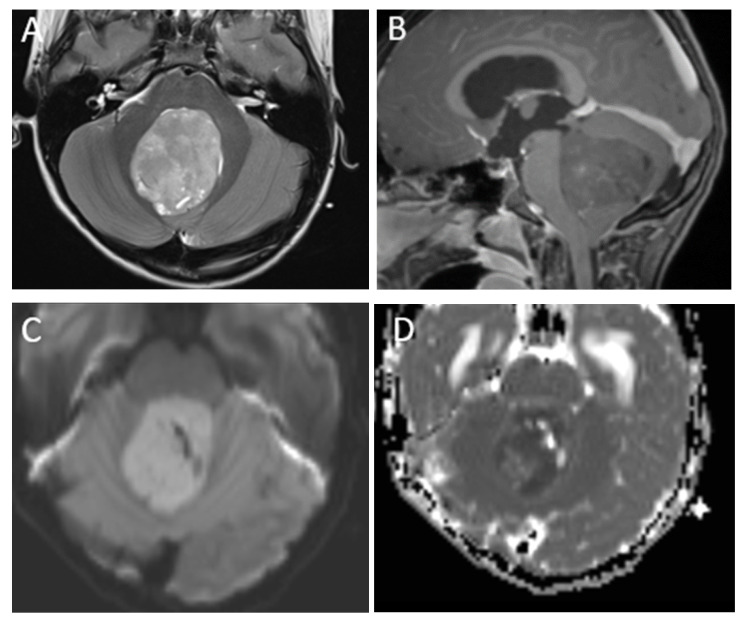

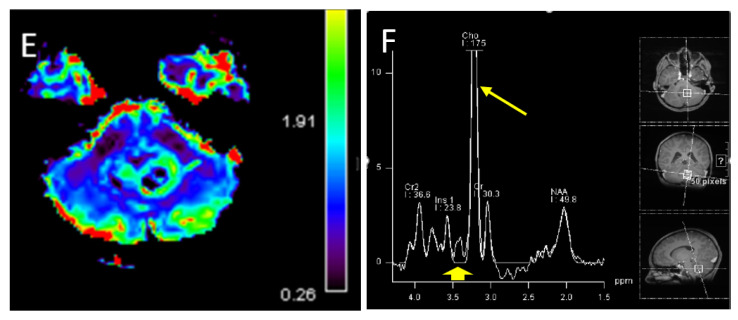
A 7-year-old boy with medulloblastoma. Axial T2 (**A**), sagittal post-contrast T1 (**B**), images demonstrate a mildly heterogeneous midline posterior fossa mass. DWI (**C**) and ADC (**D**) imaging demonstrates diffusion restriction of the lesion relative to the adjacent cerebellar parenchyma, typical of these highly cellular neoplasms. Perfusion imaging (**E**) demonstrates increased relative cerebral blood volume within the lesion and MRS (**F**) demonstrates a high choline peak (long arrow) as well as a small taurine peak (short arrow).

**Figure 2:**
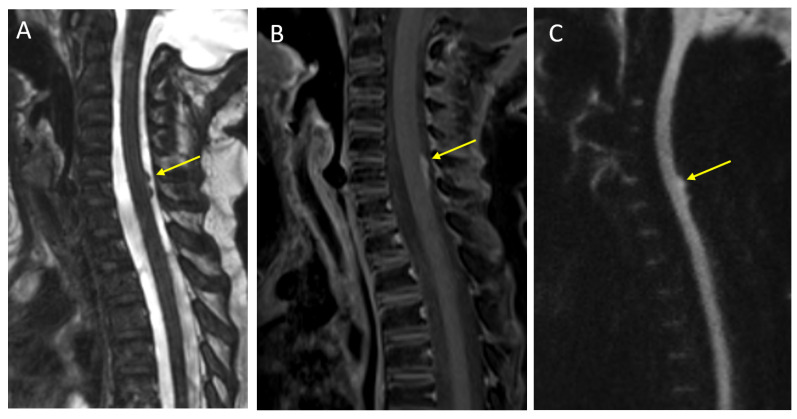
A 6-year-old girl with leptomeningeal metastasis from medulloblastoma. Sagittal CISS (**A**) and post-contrast T1 (**B**) imaging demonstrate nodular and enhancing metastatic foci along the dorsal cervical spinal cord (arrows). DWI (**C**) imaging shows associated diffusion restriction.

Perfusion imaging of medulloblastoma is variable but typically shows increased relative cerebral blood volume, as with many malignant tumors. MR spectroscopy demonstrates significantly elevated choline and reduced NAA, although this is not a specific finding and can be seen in many high-grade tumors. Taurine metabolites have been identified in some medulloblastomas and the identification of a taurine peak to the left of choline on MRS can be a more specific finding [36]. The characteristic imaging findings of medulloblastoma are illustrated in Figure 1, and leptomeningeal metastasis in the spine is illustrated in Figure 2.

Classically, medulloblastomas have been considered as midline posterior fossa lesions arising from the medullary vellum and compressing the fourth ventricle. However, these tumors are known to occur eccentrically as well. The variability in appearance and location reflects the heterogeneous molecular pathology of these tumors. Traditionally, these tumors have been classified into four histological types: classic medulloblastoma, desmoplastic/nodular medulloblastoma, medulloblastoma with extensive nodularity, and large cell/anaplastic medulloblastoma [30]. However, recently updated classifications incorporate important molecular subtypes, which more accurately predict tumor behavior, prognosis, and may guide treatment. The 2016 WHO classification introduced four distinct molecular subtypes of medulloblastoma, which have been further expanded in the most recent 2021 WHO classification. The current classification includes: WNT-activated, sonic hedgehog (SHH)-activated and *TP53* wildtype, SHH-activated and *TP53* mutant, and non-WNT/non-SHH. The SHH-activated are further divided into four subgroups and non-WNT/non-SHH into eight subgroups [3,30].

**Figure 3:**
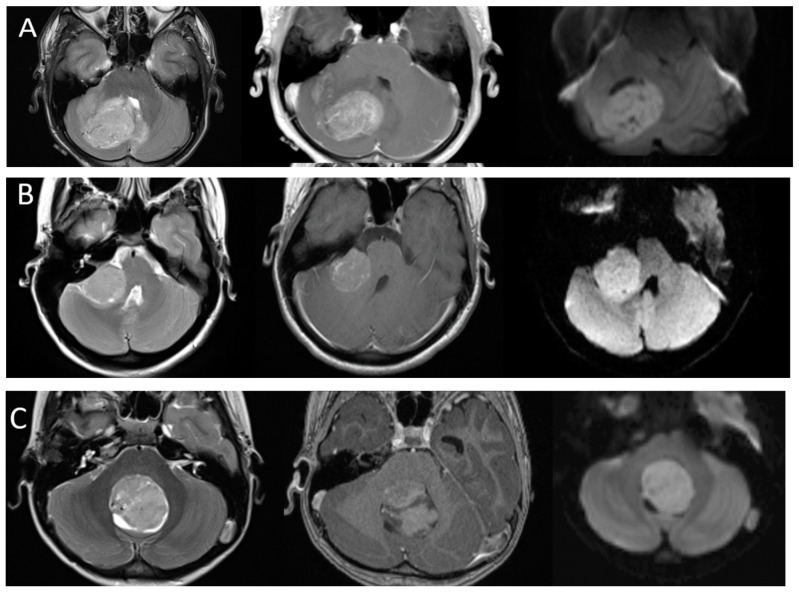

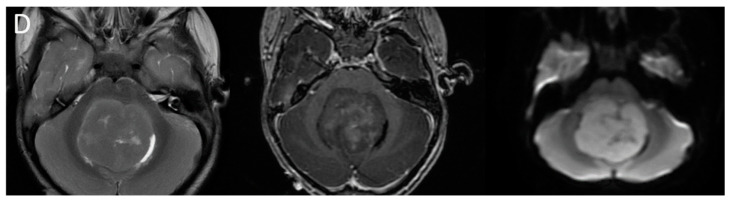
Imaging appearances of molecular subtypes of medulloblastoma. Each row includes axial T2-weighted, axial post-contrast T1-weighted and axial diffusion-weighted images from left to right. (Row (**A**)): SHH. Imaging demonstrates an off-midline lesion with the classic cerebellar hemispheric location of these lesions. (Row (**B**)): WNT. Imaging demonstrates the classic CP angle location of this subtype, although many of these lesions may arise in the midline as well. (Row (**C**)): Group 3 and (Row (**D**)): Group 4. These tumors are classically located in the midline. Note the relative hypoenhancement of the group 4 tumor, which has been a described feature. All lesions demonstrate characteristic restricted diffusion.

As the understanding of the importance of molecular and genetic profiles of these tumors increases, there has been growing interest in identifying imaging characteristics specific to the different molecular subtypes and some distinguishing features have emerged (Figure 3). Non-WNT/non-SHH tumors are the most common and are classically located in the midline, involving the fourth ventricle. Of the subgroups of non-WNT/non-SHH, group 3 tumors tend to show robust enhancement while group 4 tumors have been reported to be hypoenhancing [37]. In contrast to most medulloblastomas, group 3 tumors are often reported to have ill-defined margins [35]. WNT tumors are the least common, but have the best prognosis, and can arise near the cerebellopontine angle or in the midline. SHH tumors have intermediate prognosis, are associated with desmoplastic pathology, and are often located laterally in the cerebellar hemispheres [38]. It is important to note that while suggestive, location and imaging characteristics are not yet absolute predictors of tumor type. Machine learning approaches such as radiomics and multi-parameter imaging including MR spectroscopy have shown promise in teasing out molecular subgroups by imaging [39,40,41,42]. 

### 3.3. Atypical Teratoid/Rhabdoid Tumors

**Figure 4:**
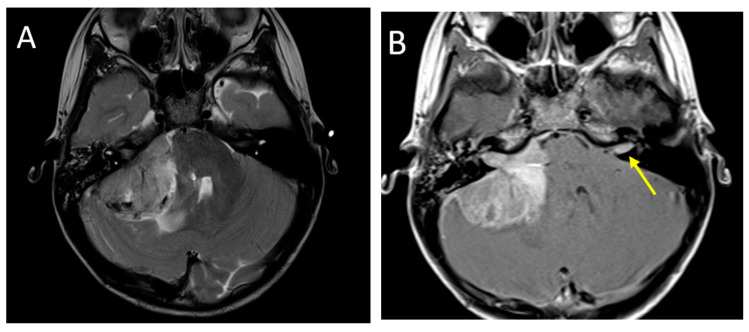

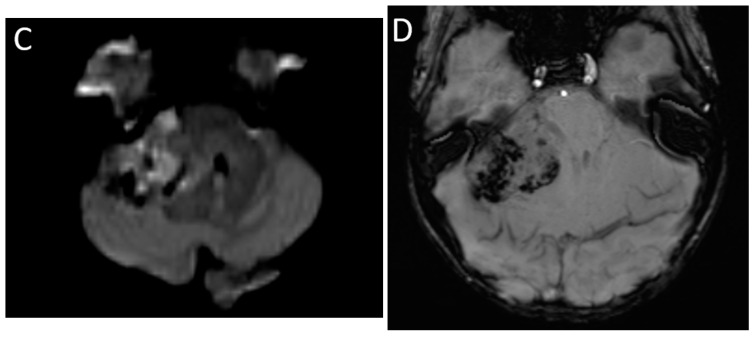
A 2.5-year-old girl with AT/RT. Axial T2 (**A**), post-contrast T1 (**B**), images demonstrate a heterogeneous lesion in the right cerebellopontine angle extending into the internal auditory canal. There is also a metastatic lesion in the left IAC (arrow). DWI (**C**) and SWI (**D**) imaging demonstrate restricted diffusion and susceptibility common in these lesions.

AT/RT is a rare, highly malignant embryonal CNS tumor occurring in young children, with the majority of cases presenting before 3 years of age [43]. These tumors can occur in both supra- and infratentorial locations, have a slight preponderance in the posterior fossa, and may be intra- or extra-axial [44]. AT/RT is an aggressive small round blue cell tumor and demonstrates imaging characteristics similar to other highly cellular tumors. As with medulloblastoma, these lesions demonstrate diffusion restriction with low signal on ADC, and the T2 signal is variable but often relatively low [35,45]. Cysts and susceptibility related to calcification or hemorrhage, are common in AT/RTs [44]. AT/RT demonstrates increased perfusion, and high choline on MRS and a high lipid peak are often encountered [45]. Myo-inositol and taurine metabolites are typically not observed. Younger age at presentation and relatively more heterogenous appearance can point towards AT/RT, although conventional MRI findings are not reliable in distinguishing AT/RT from other high-grade supra- and infratentorial tumors. Machine learning-based radiomic technique has shown promise in non-invasively distinguishing AT/RT from medulloblastoma [46]. An imaging example of posterior fossa AT/RT is presented in Figure 4. 

### 3.4. Supratentorial Embryonal Tumors

**Figure 5:**
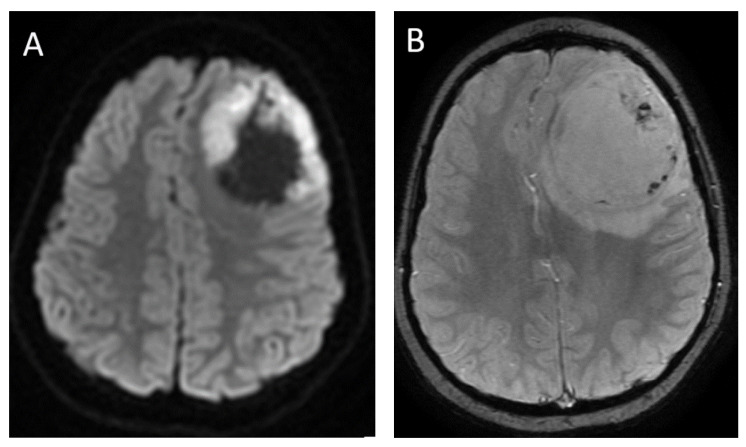

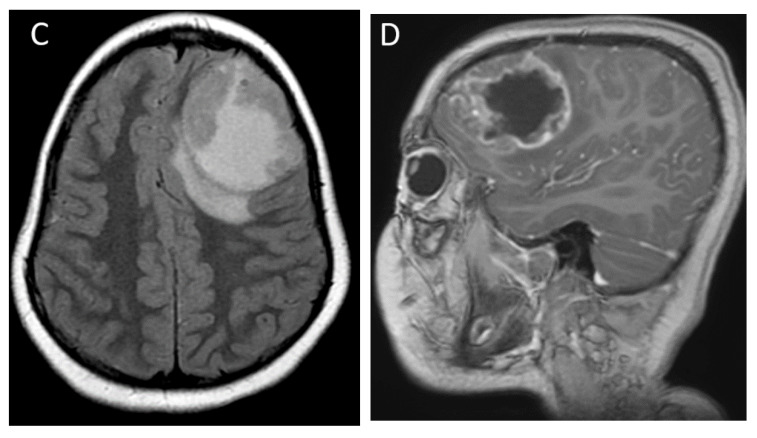
A 6-month-old male with CNS embryonal tumor, NOS, WHO Grade 4. Axial DWI (**A**), axial SWI (**B**), axial T2 FLAIR (**C**) and sagittal post-contrast T1-weighted (**D**) images demonstrate a large mass in the left frontal lobe, with diffusion restriction of the peripheral solid components, scattered small amounts of blood products and/or mineralization, isointense T2 signal of solid portion, large area of central necrosis/cyst, and heterogenous post-contrast enhancement of the solid portions. Note the similarities in appearance to the HGG illustrated in Figure 1; however, this tumor presented at a much younger age.

Supratentorial embryonal tumors represent approximately 15% of CNS neoplasms in children and are biologically distinct from medulloblastomas, their infratentorial counterparts. The classification of supratentorial embryonal tumors has rapidly evolved over the last decade, reflecting the improved understanding of the molecular and genetic make-up of these tumors [47,48]. This category includes specific subtypes, such as embryonal tumor with multilayered rosettes (ETMR); CNS neuroblastoma, FOXR2-activated; and atypical teratoid/rhabdoid tumor (AT/RT), in addition to a broader designation of CNS embryonal tumor (not otherwise specified; NOS) [3]. On MRI, these tumors are typically large with a median size of 5.7 cm and variable amounts of surrounding vasogenic edema. The solid components demonstrate diffusion restriction and post-contrast enhancement. Cystic and necrotic change of varying extent is present in the majority, and hemorrhage/calcification is seen in up to 40% of cases (Figure 5) [49]. Embryonal tumors may be indistinguishable from supratentorial high-grade gliomas and ependymomas by imaging, a distinction that is also challenging by routine histopathology and often requiring immunohistochemistry and/or molecular profiling for accurate diagnosis [49]. 

### 3.5. Pediatric Gliomas

Gliomas are overall the most common pediatric brain tumors and can have varying tumor grades. The WHO CNS5 classifies gliomas into diffuse and circumscribed types, and separates diffuse gliomas into adult and pediatric types, with further subclassification based on combined histological and molecular characteristics [3]. Key MRI features of glial tumors are summarized in Table 2.

### 3.6. Pediatric-Type Diffuse High-Grade Gliomas

High-grade gliomas constitute 8–12% of all pediatric CNS neoplasms, and one-third of these are supratentorial [50]. The current classification of pediatric-type diffuse high-grade gliomas includes: Diffuse midline glioma, H3 K27-altered; Diffuse hemispheric glioma, H3 G34-mutant; Diffuse pediatric-type high-grade glioma, H3-wildtype and IDH-wildtype; and infant-type hemispheric glioma. The previously used terms ‘anaplastic astrocytoma’ (grade 3) and ‘glioblastoma’ (grade 4) are no longer used in the context of pediatric tumors [3].

#### 3.6.1. Diffuse Midline Glioma

Diffuse midline glioma H3 K27-altered (DMG) is a specific tumor entity that involves midline locations such as pons, thalami, and spinal cord, and has a dismal prognosis. This group encompasses nearly 80% of the pontine high-grade gliomas traditionally referred to as diffuse intrinsic pontine gliomas (DIPG). The pontine gliomas are more common in younger children with a median age of 6.2 years [51]. Historically, because of the characteristic location and imaging appearance, the diagnosis of DIPG was made with imaging alone. However, given the deepening biological understanding of this disease, potential for new therapies, and increased safety of stereotactic biopsy, tissue acquisition is developing a central role in the management of these tumors [52].

**Figure 6:**
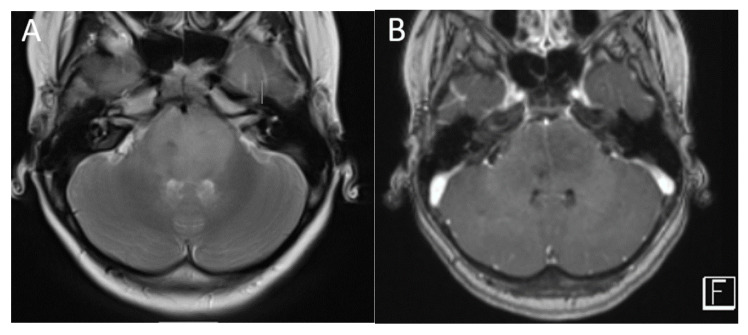

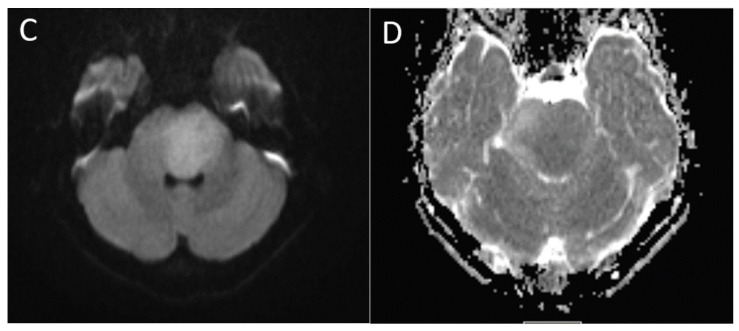
A 5-year-old boy with diffuse midline glioma H3K27-altered. Axial T2 (**A**), post-contrast T1 (**B**) images demonstrate a poorly marginated infiltrative, expansile T2 hyperintense lesion centered in the pons with little to no enhancement. DWI (**C**) and ADC (**D**) images demonstrate heterogenous signal without significant diffusion restriction.

Pontine DMG have a predilection for the ventral or basal portion of pons, and demonstrate expansile and infiltrative T2 hyperintensity, ill-defined margins, and often engulf the basilar artery without vascular narrowing. The majority of these lesions are non-enhancing at diagnosis, with patchy or ring-like enhancement seen in one-third of cases, although enhancement and necrosis are much more common after radiation treatment [53]. These lesions do not demonstrate the same degree of diffusion restriction as medulloblastoma or AT/RT; however, localized areas of relative diffusion restriction can be visually identified in up to 63% of cases [51,54]. Significant hemorrhage is uncommon at diagnosis, although small foci of internal hemorrhage may be seen in half the cases on more sensitive SWI or GRE sequences. Various conventional and quantitative imaging features have been studied as predictors of overall survival. The presence of necrosis, enhancement, and lower ADC (diffusion restriction) at diagnosis have all been correlated with worse survival chances [51]. Extrapontine extension of the tumor can be seen in up to 90% of cases and includes midbrain, thalami, internal capsules, medulla, middle cerebellar peduncles, and cerebellum. Lateral extension into the cerebellar peduncles has been associated with a worse prognosis, but not craniocaudal extension into the midbrain or medulla [55]. MRS may be variable but it typically demonstrates elevated choline with respect to NAA metabolites [13]. Metastases are rare, but they are described in about 3% of cases at diagnosis in one large series [51]. The typical imaging appearance of a pontine diffuse midline glioma is presented in Figure 6. 

In the supratentorial compartment, the diffuse midline glioma, H3 K27-altered tumors are typically centered in the thalami, often with bilateral but asymmetric involvement. The imaging features are similar to pontine midline gliomas, with hyperintensity on T2-weighted images, ill-defined, infiltrative margins, and little to no surrounding edema. Diffusion restriction, hemorrhage, and enhancement are less common at diagnosis, although patchy or ring-like enhancement associated with areas of necrosis may be present. 

#### 3.6.2. Non-Midline Diffuse High-Grade Gliomas

**Figure 7:**
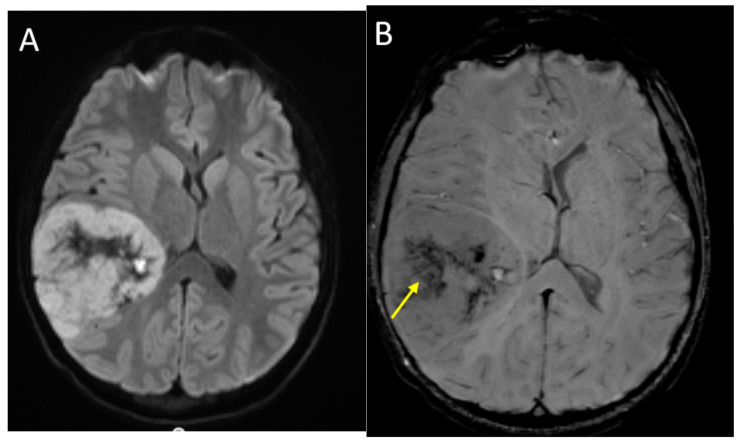

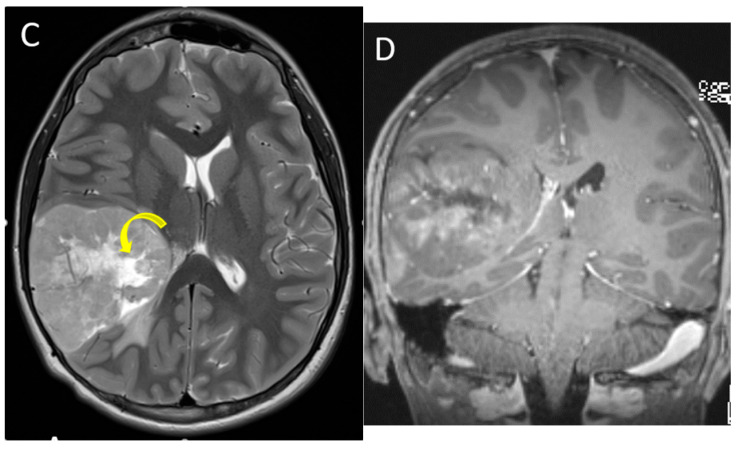
A 14-year-old male with H3 G34R mutant high-grade glioma. Axial DWI (**A**), axial SWI (**B**), axial T2 (**C**) and coronal post-contrast T1-weighted (**D**) images demonstrate a large mass in the right parieto-temporal lobes, with diffusion restriction of the solid portions, central areas of blood products and/or mineralization (arrow), isointense T2 signal with central necrosis (curved arrow), and heterogenous post-contrast enhancement.

Supratentorial, non-midline, high-grade gliomas include H3 G34 mutant and H3 wild-type, and they are most commonly seen in adolescents [50]. On imaging, these usually present as large hemispheric and/or deep gray masses with a variable degree of heterogeneity, cyst formation, hemorrhage, and calcifications. On T2-weighted images, the solid components are iso- to mildly hyperintense, and there is surrounding vasogenic edema, mass effect, and often hydrocephalus. In contrast to pontine lesions, diffusion restriction is common in the solid components, reflecting the high cellularity and high nuclear-to-cytoplasmic ratio. Post-contrast enhancement is also common, although to a variable extent within the tumor. (Figure 7) Hemispheric high-grade gliomas share these imaging features with supratentorial embryonal tumors and ependymomas, and radiological distinctions between these entities may not be possible, although HGG are usually more often seen in older children than embryonal tumors [49,50]. 

### 3.7. Pediatric-Type Diffuse Low-Grade Gliomas

Diffuse low-grade gliomas are much less common compared to circumscribed low-grade gliomas in children. Diffuse astrocytoma is the prototypical subtype, most commonly seen in the frontal or parietal lobes, and on MRI appears as a homogenously T2-hyperintense, T1-hypointense lesion with ill-defined margins [56]. These tumors generally lack diffusion restriction and post-contrast enhancement. Although in adults, diffuse astrocytomas often degenerate into higher grade, such a transformation is considered rare in children [50]. Other diffuse low-grade gliomas include angiocentric glioma; polymorphous low-grade neuroepithelial tumor of the young (PLNTY); and diffuse low-grade glioma, *MAPK* pathway-altered, with the last one encompassing tumors with an astrocytic or oligodendroglial morphology [3].

### 3.8. Circumscribed Astrocytic Gliomas

The most common circumscribed glioma is pilocytic astrocytoma, which accounts for one-third of all gliomas and is the most common intracranial neoplasm in children 0–14 years of age [57]. It is a benign, grade 1 tumor that has excellent prognosis with resection only. The tumorigenesis is related to alteration in *MAPK* pathway, commonly as *BRAF* fusion or *BRAF V600E* point mutation, and can also be seen in association with neurofibromatosis-type 1 [58].

**Figure 8:**
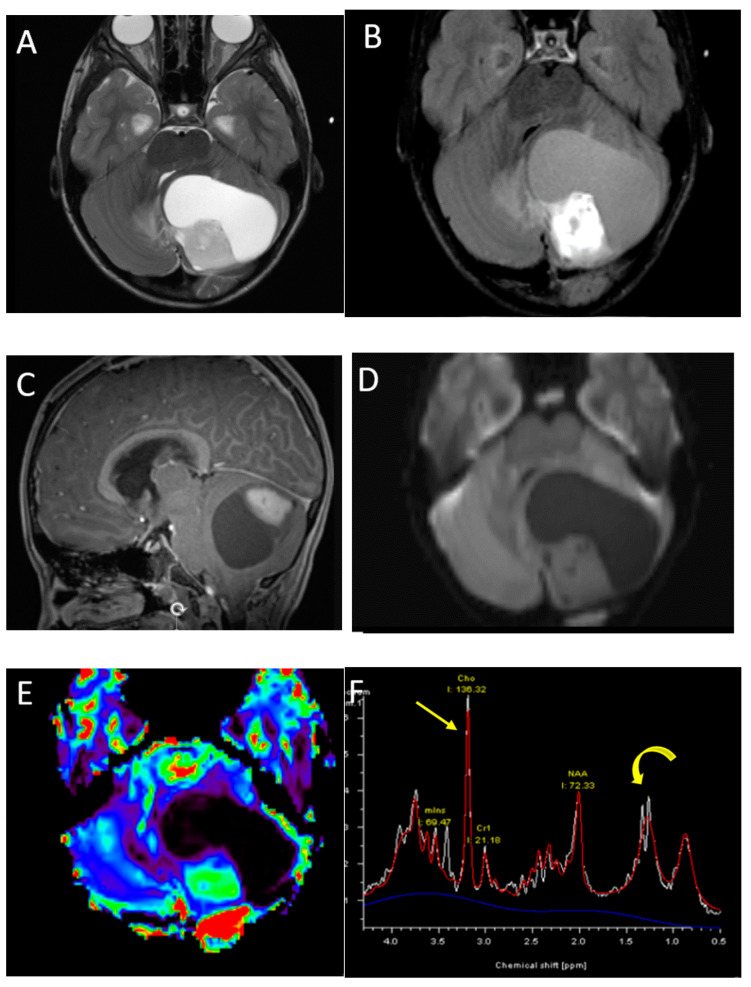
A 6-year-old boy with pilocytic astrocytoma. Axial T2 (**A**), post-contrast T2-FLAIR (**B**) sagittal post-contrast T1 (**C**) and axial DWI (**D**) images demonstrate a characteristic large cystic lesion in the left cerebellar hemisphere with a solid nodule. The nodule enhances intensely without significant diffusion restriction compared with adjacent parenchyma. Perfusion imaging (**E**) demonstrates significantly increased perfusion of the solid nodule. MRS (**F**) shows elevated choline (arrow) and high lipid lactate peaks (curved arrow).

Pilocytic astrocytomas are most often encountered in the posterior fossa [35] as intra-axial lesions, classically arising from the cerebellar hemispheres. The typical imaging appearance is a well-circumscribed cystic lesion with a solid, mural nodule. However, other presentations including heterogeneously solid, multicystic, and occasionally hemorrhagic masses may be encountered [59]. The cystic component demonstrates hyperintense, near-fluid signal on T2 and increased diffusivity, but it may have a variable signal on T2 FLAIR imaging due to proteinaceous contents. The solid component often demonstrates robust enhancement and there is often enhancement of the cyst wall. The solid component does not restrict diffusion, with reported ADC values of 1.7–1.8 × 10^−3^ mm/s [2], which is a distinguishing feature from high-grade tumors such as medulloblastoma [33,60]. On MR perfusion, there is often increased relative cerebral blood volume in the solid component, and on MRS there may be significantly elevated choline, despite the low-grade nature of the tumor [61]. High lipid/lactate peaks are common in JPAs [13]. The classic imaging appearance of a posterior fossa JPA is shown in Figure 8. Metastasis at presentation or recurrence is rare in in posterior fossa JPAs although spine imaging at diagnosis should be considered, as the histology may be uncertain pre-operatively [62]. 

Supratentorial pilocytic astrocytomas are overall less common than their posterior fossa counterparts, and most frequently involve the optic pathway–hypothalamic axis. On MRI, these tumors present as expansile T2 hyperintense masses, typically centered in the optic chiasm, with variable involvement of the optic nerves, optic tracts, lateral geniculate bodies, hypothalamus and third ventricle. Post-contrast enhancement is often present but variable [63]. Therapeutic decisions are generally based on changes in tumor size (which can be assessed on T2-weighted images) and ophthalmic examination, with isolated changes in tumor enhancement considered less important. The suggestion has been made to forego post-contrast imaging in routine surveillance of optic pathway gliomas to decrease the risk of gadolinium deposition and to also decrease imaging time and costs [64,65].

Also belonging to the category of circumscribed astrocytomas are pleomorphic xanthoastrocytomas, which are rare astrocytic neoplasms of childhood and young adulthood. Most of these are WHO grade 2 and have a favorable prognosis, although 10–23% may have a more aggressive behavior [66]. These tumors are most commonly seen in the temporal or frontal lobes, are peripheral in location, abutting the meninges, and often scallop the inner table of the calvarium because of slow growth. The dominant cystic component is seen in two-thirds of cases and surrounding vasogenic edema is common. The solid components display avid but heterogenous enhancement, and diffusion restriction is usually absent [67,68].

### 3.9. Ependymoma

Ependymomas constitute 10% of all primary CNS neoplasms in children, and although slightly more common in the posterior fossa, approximately 40% are supratentorial [50]. The supratentorial, infratentorial, and spinal ependymomas are considered genetically distinct and are now classified separately by WHO CNS5 based on specific molecular alterations [3].

#### 3.9.1. Infratentorial Ependymomas

**Figure 9:**
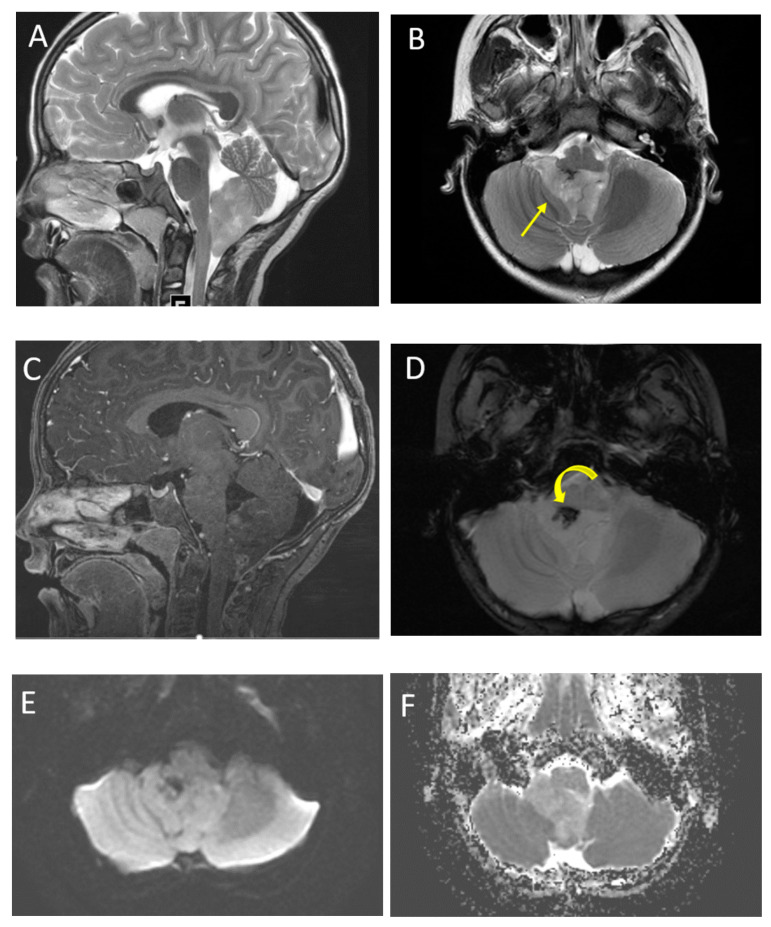
A 6-year-old male with ependymoma. Sagittal and axial T2 images (**A**,**B**) demonstrate a heterogeneous lesion involving the fourth ventricle. Note the characteristic extension through the foramen of Luska (arrow). Post-contrast T1 imaging (**C**) demonstrates typical heterogeneous enhancement. SWI imaging (**D**) demonstrates a focus of low signal correlating with calcification (curved arrow). DWI (**E**) and ADC (**F**) imaging show increased ADC values of the tumor compared with adjacent parenchyma.

In the posterior fossa, ependymomas are most commonly encountered in the region of the fourth ventricle [35]. In comparison to medulloblastoma, ependymomas have the appearance of pliable tumors that characteristically extend through the foramina of Luschka and/or Magendie, encasing nerve roots and vascular structures. These lesions are typically T2 hyperintense and demonstrate variable enhancement, although the signal characteristics can be heterogenous. Cysts are common and often larger than seen in medulloblastomas. Calcification is also common, occurring in approximately 50% of lesions, and are better characterized in CT than MRI [69]. These lesions are less cellular than medulloblastomas, typically grade 2 or 3, and classically do not have the same degree of diffusion restriction, with a reported mean ADC of approximately 1.1 [33]. However, greater degrees of diffusion restriction may occasionally be encountered, likely reflecting higher grade or anaplastic lesions, and DWI values overlapping with medulloblastomas have been reported [70]. The typical imaging appearance of a posterior fossa ependymoma is demonstrated in Figure 9. 

Two molecular subgroups of posterior fossa ependymoma have been identified: posterior fossa A (PFA) and posterior fossa B (PFB) [3]. PFB lesions typically originate from the floor of the fourth ventricle more inferiorly toward the obex, seen in older children and adolescents, and have an excellent prognosis. PFA lesions more commonly arise along the lateral recess and may extend into the prepontine recess, making complete resection difficult. PFA lesions are predominantly seen in very young children, and portend poor prognosis [58].

Similar to other CNS neoplasms, ependymomas typically demonstrate increased relative cerebral blood volume in perfusion imaging, and high choline levels and reduced NAA in spectroscopy. Ependymomas often demonstrate significantly elevated myo-inositol metabolites (to the left of the choline peak) and lipid/lactate peaks may be present [71].

Although not as common as in medulloblastomas, leptomeningeal metastasis is seen in an estimated 3–10% of cases at presentation [72]. More frequently, metastases occur during disease recurrence [69] and, as with medulloblastoma, these patients require brain and whole spine imaging for disease workup and surveillance.

#### 3.9.2. Supratentorial Ependymomas

The majority of supratentorial ependymomas are parenchymal in location, with a predilection for the frontal and parietal lobes, although a small fraction can be intraventricular [50,73]. These are typically large masses, with both solid and cystic components, and surrounding vasogenic edema. Central or centripetal, chunky calcifications are common, and there may be areas of necrosis within the solid component. Enhancement is usually present but heterogenous, and diffusion restriction may be seen in two-thirds of cases [73]. The differential considerations for parenchymal lesions include high-grade glioma and embryonal tumor, while intraventricular locations include choroid plexus tumors and giant cell astrocytoma. Of the two molecular subtypes of supratentorial EP, *ZFTA* fusion-positive usually occur in older children and have a poor prognosis compared with the *YAP1* fusion-positive, which are more typical in infants and associated with a very good prognosis [58].

### 3.10. Glioneuronal and Neuronal Tumors

These are rare tumors, accounting for 1% of pediatric brain tumors and usually seen in older children and adolescents. The most common tumors in this category are ganglioglioma and dysembryoplastic neuroepithelial tumor (DNET). Key MRI features of glial and glioneuronal tumors are summarized in Table 2.

**Figure 10:**
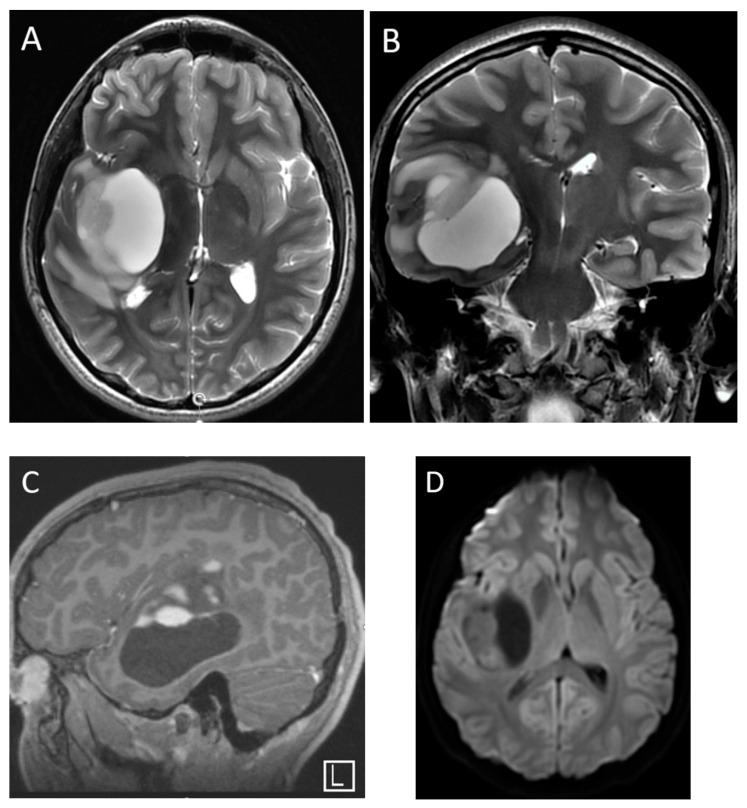
A 16-year-old male with ganglioglioma, presenting with seizures. Axial and coronal T2-weighted (**A**,**B**), sagittal post-contrast T1-weighted (**C**) and axial DWI (**D**) images demonstrate a large predominantly cystic mass with eccentric solid component in the right temporal lobe. There is patchy but avid enhancement within the solid component. Lack of diffusion restriction within the solid component helps distinguish this from higher grade tumors.

Gangliogliomas are composed of neoplastic neuronal elements and astrocytes, have an indolent course and excellent prognosis. Over three-fourths are seen in the temporal lobes, particularly mesial temporal lobes, and the patients typically present with seizures. On MRI, the appearance can be variable, although 40% tumors present as mixed solid and cystic masses, with an avidly enhancing mural nodule. Calcifications are common and diffusion restriction is absent [50,56] (Figure 10).

DNETs are cortically based benign tumors, presenting with seizures, and 30% have an adjacent cortical dysplasia. On MRI, they are well-circumscribed, may have a triangular configuration, broader at the cortex and tapering towards the ventricles. The tumors may display small cysts and bubbly appearance, and often there is scalloping of the overlying calvarium. DNETs typically do not enhance, although small nodular or ring-like foci of enhancement may be seen in one-third of cases [56,74].

### 3.11. Choroid Plexus Tumors

**Figure 11:**
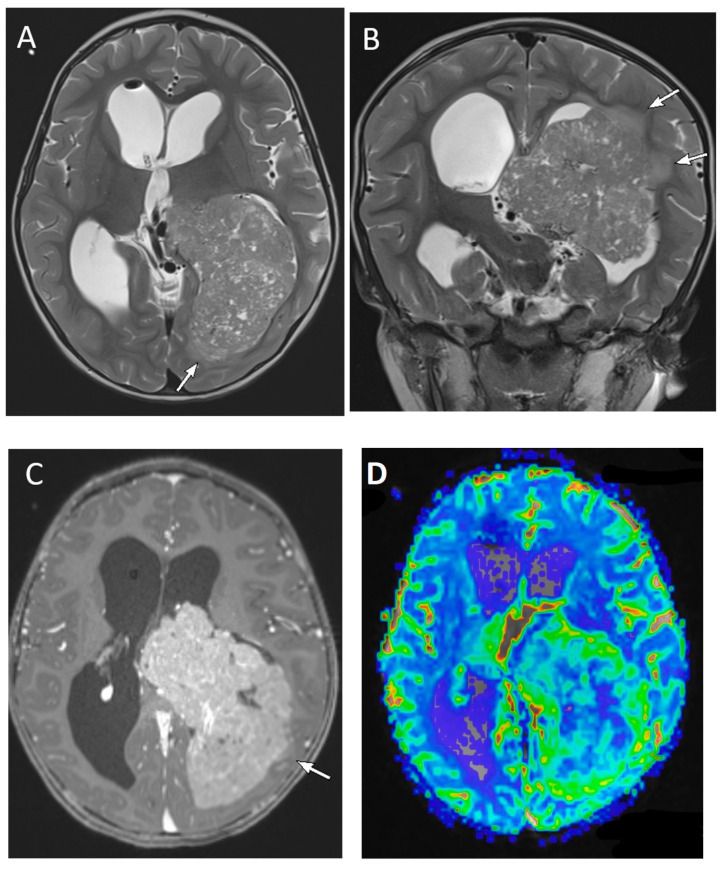
A 2-year-old male with choroid plexus carcinoma. Axial and coronal T2-weighted (**A**,**B**), axial post-contrast T1-weighted (**C**) and axial perfusion rCBF (**D**) images demonstrate a large, predominantly solid mass with scattered tiny cystic foci, within the atrium and occipital horn of the left lateral ventricle. There is diffuse enhancement of the tumor and increased blood flow on the perfusion maps. The presence of parenchymal invasion (arrows on (**A**–**C**)) distinguishes choroid plexus carcinoma from papilloma.

Choroid plexus tumors are rare, accounting for 2–4% of brain tumors in children. These are most commonly located in the lateral ventricles, followed by the fourth and the third ventricles. The mean age at presentation is 4.6 years. Both choroid plexus papillomas and carcinomas appear as papillary or lobulated, enhancing, intraventricular masses, although carcinomas are more likely to demonstrate internal heterogeneity, necrosis, parenchymal invasion, and perilesional edema (Figure 11). Hydrocephalus is often present and may be secondary to CSF overproduction and/or obstruction, depending on the location and tumor differentiation [75,76]. On MRS, choroid plexus papillomas demonstrate higher myo-inositol and lower choline peaks compared to carcinomas [12]. Key MRI features of choroid plexus tumors are summarized in Table 3.

### 3.12. Pineal Region Tumors

Pineal region tumors account for approximately 4% of childhood intracranial tumors and include germ cell tumors and pineal parenchymal tumors, which are often difficult to distinguish by imaging. Key MRI features of pineal region tumors are summarized in Table 3.

**Figure 12:**
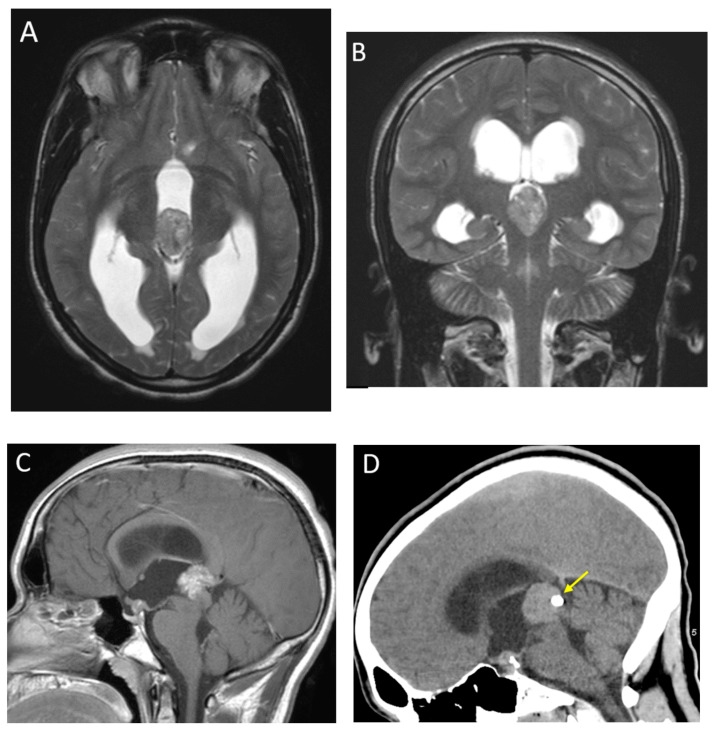
A 19-month-old male with with germ cell tumor and synchronous involvement of the pineal and suprasellar regions. Axial and coronal T2-weighted (**A**,**B**) images of the pineal region demonstrate heterogeneously T2-isointense mass with obstructive hydrocephalus. Sagittal post-contrast T1-weighted image (**C**) demonstrates heterogenous enhancement of the pineal region mass with obstruction of the cerebral aqueduct, and also a smaller, similar-appearing mass, involving the pituitary infundibulum (arrowhead). Sagittal non-contrast CT image (**D**) of the same patient demonstrates a rounded pineal calcification ‘engulfed’ by the mass (arrow), which can help to distinguish germ cell tumors from pineoblastomas.

Germ cell tumors are relatively more common, typically seen in adolescent males, and include germinomas and non-germinomatous germ cell tumors. Serum and CSF oncoprotein markers, such as alpha-fetoprotein, beta-human chorionic gonadotropin, and placental alkaline phosphatase, are helpful in diagnosis and follow-up. Pineal parenchymal tumors include pineocytomas, pineal parenchymal tumors of intermediate differentiation, and pineoblastomas. Of these, pineoblastomas are the most common, accounting for 40% of pineal parenchymal tumors. Pineoblastomas are WHO grade 4, undifferentiated tumors, that are prone to adjacent parenchymal invasion and CSF dissemination [77]. Both germ cell tumors and pineoblastomas can have heterogenous signal and enhancement with diffusion restriction. Apparent diffusion coefficient (ADC) values have been used to distinguish between the two, with mixed results. The relationship between the tumor and the pattern of pineal gland calcification on CT can sometimes help in diagnosis, with germinomas typically ‘engulfing’ the calcification, and pineoblastomas ‘exploding’ the calcification to the periphery of the mass. Germinomas can also involve the pituitary infundibulum, either in isolation or concurrent with pineal involvement (Figure 12). Pineoblastomas can be associated with retinoblastomas as a part of ‘trilateral’ disease in a small percentage of patients [78]. 

## 4. Conclusions

MRI plays a key role in the diagnosis and treatment of pediatric brain tumors. This review provides an overview of conventional and advanced MRI imaging techniques and the key imaging features of major intracranial tumors, a basic understanding of which can be very helpful in the management of these children.

## Figures and Tables

**Table 1:** Summary of key imaging features of embryonal tumors.

Tumor Type	Common Location	Key MRI Features
Medulloblastoma	Exclusively posterior fossa Most commonly in fourth ventricle/cerebellar vermis (non-WNT, non-SHH, or WNT), can involve cerebellopontine angle (WNT) or cerebellar hemispheres with extra-axial extension (SHH)	Diffusion restricting Variable enhancement Cystic/necrotic change may be present Calcifications uncommon Taurine peak characteristic
Atypical teratoid/rhabdoid tumor	Posterior fossa (slightly more common) or cerebral hemispheres May be extra-axial	Diffusion restricting Enhancement usually present More heterogenous than medulloblastomas, with cysts/necrosis, calcification, and hemorrhage
Supratentorial embryonal	Cerebral hemispheres or deep nuclei, rarely intraventricular	Large tumor Diffusion restricting solid components Variable cysts/necrosis and hemorrhage

**Table 2:** Summary of key imaging features of glial and glioneuronal tumors.

Tumor Type	Common Location	Key MRI Features
Diffuse high-grade gliomas		
Diffuse midline glioma (H3K27 altered)	Ventral pons and thalami	Expansile, ill-defined mass No diffusion restriction Usually, non-enhancing at presentation Encasement of basilar artery without narrowing
Supratentorial high-grade glioma (H3 G34 mutant or H3 wild-type)	Hemispheric or deep nuclei; most common in frontal and parietal lobes	Large, circumscribed tumor Diffusion restricting solid components Variable cysts/necrosis and hemorrhage
Circumscribed gliomas		
Pilocytic astrocytoma	Cerebellum (most common), brainstem, optic chiasm/hypothalamus	Most commonly, cyst with enhancing mural nodule although cystic component can be variable, and may be completely solid
Ependymoma		
Posterior fossa ependymoma	Fourth ventricle (PFB) or cerebellopontine angle (PFA)	Heterogeneous mass Calcifications common Intermediate diffusion (between medulloblastoma and pilocytic astrocytoma) Usually enhancing High myo-inositol on MRS
Supratentorial ependymoma	Frontal or parietal parenchyma	Large mass with necrosis Central chunky calcifications Diffusion restriction in two-thirds
Neuronal and glioneuronal tumors		
Ganglioglioma	Temporal lobe	Variable, although commonly cystic with enhancing mural nodule No diffusion restriction
Dysembryoplastic neuroepithelial tumor (DNET)	Frontal or temporal lobes, cortically based	Well-circumscribed, triangular configuration, ‘bubbly’ appearance Absent or minimal enhancement May be associated with cortical dysplasia Scalloping of the overlying calvarium

(Abbreviations: rCBV, relative cerebral blood volume; MRS, Magnetic Resonance Spectroscopy; NAA, N-acetylaspartate; PFA, posterior fossa A; PFB, posterior fossa B).

**Table 3:** Summary of key imaging features of pineal region and choroid plexus tumors.

Tumor Type	Common Location	Key MRI Features
Pineal region tumors		
Pineoblastoma	Pineal region	Heterogenous, ‘explode’ pineal calcification Diffusion restricting Enhancement variable
Germ cell tumor	Pineal and/or suprasellar region	Heterogenous, usually calcified, ‘engulf’ pineal calcification Diffusion restricting Enhancement usually present
Choroid plexus tumors		
Choroid plexus papilloma	Lateral ventricle followed by fourth ventricle	Lobular or papillary lesions Diffuse enhancement No diffusion restriction High myo-inositol, low creatine, moderate choline on MRS
Choroid plexus carcinoma	Lateral ventricle	More heterogenous than papillomas Parenchymal invasion/edema May have diffusion restriction Moderate myo-inositol and high choline on MRS
